# Neuralgia of Jaw Following Tooth Extraction

**Published:** 1871-05

**Authors:** 


### ARTICLE X.

### *Neuralgia of Jaw following Tooth Extraction; Treatment by Prof. Gross' Method.*

In the number of the *American Journal of the Medical Sbiences*, for July, 1870, is a description by Professor Gross, of a form of neuralgia of the jaw-bones, following tooth extraction, and depending as he thinks, on compression of the alveolar nerves by the osseous material deposited during the reparative process. The following is an example of the affection.

Solomon K., aged 18, had for some weeks been attending as an out-patient at the Leeds Infirmary, on account of excruciating pain in the lower jaw. Six months ago, the left lower anterior bicuspid tooth was removed in a state of decay. In from a week to ten days afterwards, he began to suffer intense pain in the part, and occasionally a little blood oozed from the gum. He sought relief from many sources, but in vain; and during his attendance as an out patient at the infirmary, the usual anti-neuralgic remedies had been prescribed to no purpose. The affected gum was hard, and adherent to the bone; and pressure upon it aggravated the pain, which though chiefly felt where the tooth had been, radiated to a considerable distance around. After having seen Dr. Gross' statement, Mr. Jessup admitted the young man; and, on October 20th, he cut down upon and removed with bone-forceps and gouge a portion of the alveolar border. The relief was complete, and continued up to October 29th, on which day he was discharged. He has not since been seen.—*British Medical Journal*,.

